# Berberine Attenuates Cadmium-Induced Nephrotoxicity by Suppressing LDHA-Mediated Glycolytic Reprogramming and Restoring Mitochondrial TCA Cycle Metabolism

**DOI:** 10.3390/biom16070951

**Published:** 2026-06-26

**Authors:** Zikang Zeng, Weidong Qiao, Yuanyuan Zhang, Shusheng Tang

**Affiliations:** 1 Technology Innovation Center for Food Safety Surveillance and Detection (Hainan), Sanya Institute of China Agricultural University, Sanya 572025, China; 2 State Key Laboratory of Veterinary Public Health and Safety, College of Veterinary Medicine, China Agricultural University, Beijing 100193, China

**Keywords:** berberine, cadmium, nephrotoxicity, TCA cycle, LDHA, metabolomics

## Abstract

Cadmium (Cd) is an environmental nephrotoxicant that preferentially accumulates in the kidney and disrupts redox and energy metabolism. However, the protective effect of berberine (Ber) against Cd-induced nephrotoxicity remains insufficiently characterized. In the present study, male C57BL/6 mice were orally exposed to CdSO_4_ (30 mg/kg body weight/day) for 30 days in the absence or presence of berberine (25 or 100 mg/kg/day). Renal function, histopathology, oxidative stress parameters, LC–MS/MS-based metabolomic profiling, gene and protein expression, and in silico ligand–target interactions were evaluated. Cd exposure markedly increased serum CREA, renal index, renal LDH activity, and MDA content, decreased SOD and CAT activities, and induced pronounced renal histopathological lesions. Ber significantly attenuated these abnormalities in a dose-dependent manner. Metabolomic analysis revealed that Cd broadly suppressed pyruvate metabolism, tricarboxylic acid cycle intermediates, and NAD+/NADH homeostasis, whereas berberine restored the levels of pyruvate, acetyl-CoA, oxaloacetate, citrate, isocitrate, succinate, fumarate, malate, NAD+, and NADH. In parallel, berberine normalized the expression of metabolism-related genes including the downregulation of *Ldha* and the upregulation of *Cs*, *Sucnr1*, *G6pc*, and *Pfkm*, with the high-dose regimen showing the most evident recovery. Western blotting further verified the lower LDHA protein expression after berberine treatment. Molecular docking demonstrated favorable potential berberine–LDHA binding, and molecular dynamics simulation supported the stability of the ligand–protein complex. Collectively, these findings indicate that berberine ameliorates Cd-induced renal injury, an effect that correlates with attenuated oxidative stress, modulation of LDHA-associated glycolytic pathways, and restoration of mitochondrial TCA-cycle activity and redox balance, highlighting berberine as a promising candidate for the prevention of heavy metal-associated nephrotoxicity.

## 1. Introduction

Heavy metals are metallic elements characterized by relatively high atomic weights and densities, including lead, mercury, cadmium, arsenic, and chromium [1,2]. While these elements naturally occur in the environment, human activities—including industrial production, agricultural practices, and urbanization—have significantly intensified heavy metal pollution. Heavy metals are persistent environmental contaminants that accumulate over time and can enter the human body through the food chain, posing significant risks to human health [3].

Cd typically exists in the environment as a chemical compound and is naturally found in low concentrations that do not pose a health risk. It commonly co-occurs with zinc and lead in mineral deposits [4]. Cd is ranked first among the “Twelve Priority Hazardous Substances” identified by the United Nations Environment Program (UNEPs) and is classified by the International Agency for Research on Cancer (IARC) as a Group 1 carcinogen [5,6]. Environmental exposure leads to Cd accumulation in the human body and chronic toxicity. The kidney is the primary target organ for cadmium toxicity. Cadmium is carried by the bloodstream to the liver, where it induces hepatic synthesis of metallothionein (MT) in hepatocytes, forming a cadmium–metallothionein (Cd-MT) complex once exposed. Then, it is released into the circulation and gradually redistributed to other organs, primarily the kidneys. Following glomerular filtration, the Cd–MT complex is reabsorbed by proximal tubular epithelial cells, where it rapidly dissociates, liberating free Cd^2+^ that stimulates de novo MT synthesis. However, when cadmium exposure exceeds the binding capacity of MT, excess unbound Cd^2+^ accumulates within tubular cells, causing cellular injury and impairing tubular reabsorptive function, clinically manifesting as proteinuria and related kidney dysfunction [7,8,9].

Cd is a potent inducer of oxidative stress. It elevates reactive oxygen species (ROS) levels by activating NADPH oxidase and mitochondrial ROS generation pathways, often exceeding the capacity of intrinsic antioxidant systems [10,11]. Additionally, Cd interferes with gene expression, disrupts protein translation and signal transduction pathways, and triggers multiple forms of cell death. Its toxic mechanisms encompass early-response gene activation, endoplasmic reticulum stress, increased ROS production, Nrf2 antioxidant signaling pathway suppression, and mitochondrial dysfunction [12,13,14].

Berberine (Ber) is a quaternary ammonium isoquinoline alkaloid extracted from the rhizomes of plants such as *Coptis chinensis*, and its hydrochloride salt is commonly used in clinical practice [15]. Originally used as an over-the-counter antidiarrheal agent, Ber has now been shown by modern pharmacological research to exhibit multi-target activities. Specifically, it activates the AMPK (adenosine monophosphate-activated protein kinase) pathway, leading to significant improvements in insulin resistance, increased peripheral glucose uptake, and suppression of hepatic gluconeogenesis. These effects collectively mimic the antihyperglycemic action of metformin [16]. In addition, Ber can upregulate hepatic low-density lipoprotein receptor (LDLR) expression and inhibit HMG-CoA reductase, achieving a cholesterol-lowering effect synergistic with statins [17]. Ber exerts multifaceted cardiovascular protection by: (1) modulating myocardial ion channels to prevent arrhythmias, (2) attenuating fibrosis and oxidative stress to improve heart failure outcomes, and (3) inhibiting vascular smooth muscle cell proliferation—potentially decelerating the atherosclerosis progression [18]. Moreover, Ber exhibits antiplatelet aggregation, broad-spectrum anti-inflammatory effects via inhibition of the NF-κB pathway, and beneficial modulation of the gut microbiota [19,20,21]. Based on these mechanisms, Ber has emerged as an essential natural candidate for preventing and treating metabolic syndrome and cardiovascular diseases. With ongoing mechanistic research and clinical application, its value as a multi-target therapeutic agent is poised to expand further; however, its protective effects against Cd-induced nephrotoxicity remain unexplored. Given the urgent need for effective interventions against heavy metal-related renal injury, clarifying the Ber mechanisms in Cd-induced nephrotoxicity is of considerable significance. It may open new ways for therapeutic development.

## 2. Materials and Methods

### 2.1. Chemicals and Reagents

CdSO_4_ was purchased from Shanghai Yuanye Bio-technology Co., Ltd. (Shanghai, China). Ber was purchased from Shanghai Macklin Biochemical Co., Ltd. (Shanghai, China). DMSO, CMC-Na, 1% penicillin-streptomycin solution were purchased from Beijing Solarbio Science and Technology Co., Ltd. (Beijing, China).

### 2.2. Animal Treatment

All procedures involving animals were reviewed and approved by the institutional animal care and use committee of China Agricultural University in accordance with ethical guidelines (No. CAU20190901–1). Male C57BL/6 mice (6–8 weeks old, 18–22 g) were obtained from Vital River Laboratory Animal Technology Co., Ltd. (Beijing, China). Mice were allowed a one-week adaptive phase before the commencement of treatment. During experimentation, a standard environmental condition was maintained (relative humidity: 50% to 60%; room temperature: 20 °C to 25 °C; and light-dark cycle: 8:00 a.m.–8:00 p.m.).

Mice were randomly divided into control, Cd model, Cd with Berberine low-dose group, Cd with Berberine high-dose group and berberine-only group, n = 6 in each group. The detailed treatments were shown as follows:

(1) Control group: 0.5% (*w*/*v*) CMC-Na solution was administered orally daily for 30 consecutive days;
(2) Model group: Cd (30 mg/kg BW/day) was dissolved in 0.5% (*w*/*v*) CMC-Na solution and administered orally for 30 consecutive days;
(3) Low-dose combination: Ber (25 mg/kg BW/day) and Cd (30 mg/kg BW/day) were dissolved in 0.5% (*w*/*v*) CMC-Na solution and administered orally for 30 consecutive days;
(4) High-dose combination: Ber (100 mg/kg BW/day) and Cd (30 mg/kg BW/day) were dissolved in 0.5% (*w*/*v*) CMC-Na solution and administered orally for 30 consecutive days;
(5) Berberine-only group: Ber (100 mg/kg BW/day) was dissolved in 0.5% (*w*/*v*) CMC-Na solution and administered orally for 30 consecutive days.

### 2.3. Measurement of Creatinine (CREA)

Gather the fresh blood of each mouse. Then, centrifuge the sample at 3500 rpm for 10 min, 25 °C. The serum CREA levels of each sample were tested using Mindray BS-600 (Shenzhen, Guangdong, China).

### 2.4. Histopathological Analysis

Tissue samples were fixed in 10% formalin, dehydrated through a graded ethanol series, cleared in xylene, and embedded in paraffin at 60 °C. Sections (4 μm) were cut using a microtome, floated on a 45 °C water bath, mounted on glass slides, and dried at 60 °C. After deparaffinization and rehydration, the sections were stained with Harris hematoxylin, differentiated in 1% hydrochloric acid ethanol, blued in running water, and counterstained with 0.5% eosin. Finally, the slides were dehydrated, cleared, mounted with neutral resin, and examined microscopically for photodocumentation.

### 2.5. Metabolomics Analysis

Kidney tissues were homogenized in precooled medium (−80 °C, 20% methanol) and centrifuged at 12,000 rpm for 10 min at 4 °C. The supernatants were collected and vacuum freeze-dried. The dried extracts were reconstituted in acetonitrile–water (1:1, *v*/*v*, containing 0.1% formic acid), filtered through 0.22 μm membranes, transferred to vials, and stored at −80 °C until analysis. Metabolomic profiling was performed on an AB SCIEX 5500 LC–MS/MS system (AB Sciex LLC., Framingham, MA, USA). The raw peak areas obtained from LC–MS analysis were normalized to the total protein concentration of each tissue lysate, as determined by the BCA assay, to correct for variations in tissue biopsy weight and extraction efficiency. Data processing and statistical analysis were conducted using MetaboAnalyst 6.0 (https://www.metaboanalyst.ca/, accessed on 1 September 2025).

### 2.6. Western Blotting

The expression of the targeted protein was analyzed by Western blotting. In summary, the kidney tissue of each sample was homogenized using an ice-cold RIPA buffer and various protein inhibitors. An automatic low-temperature crusher from Servicebio Technology Co., Ltd. (Wuhan, China) was used to homogenize the samples, then centrifuged at 12,000 rpm for 10 min at 4 °C using a refrigerated centrifuge. The BCA protein assay kit from Accurate Biotechnology (Hunan) Co., Ltd. (Changsha, Hunan, China) was used to normalize the samples, with 20 μg of protein loaded per well. Primary antibodies were used, including LDHA and β-actin, which were obtained from Wuhan ABclonal Biotechnology Co., Ltd. (Wuhan, China), diluted 1:1000. The Tanon Chemiluminescent Imaging System (Shanghai, China) was used to visualize the blots. The protein expression in each gel was analyzed using ImageJ 1.54 from NIH (Bethesda, MD, USA).

### 2.7. Molecular Docking and Molecular Dynamics Simulation

The molecular structure of Ber was retrieved from PubChem (https://pubchem.ncbi.nlm.nih.gov/), and the LDHA protein structure (PDB ID: 1i10) was obtained from UniProtKB (https://www.uniprot.org/uniprotkb (accessed on 1 September 2025). Molecular docking was performed using Dockey v1.0.3 to evaluate the binding affinity and interaction modes between Ber and LDHA. Binding poses and intermolecular interactions were visualized with Discovery Studio 2019 Client. Molecular dynamics (MD) simulations were conducted with GROMACS from the University of Groningen (Groningen, the Netherlands). Root mean square deviation (RMSD) analysis was conducted by aligning protein backbone atoms to the initial structure and calculating structural deviations over a 100 ns trajectory.

### 2.8. Statistical Analysis

Differential metabolites were identified using combined criteria as follows: fold change >1 (or <1 for down-regulation), adjusted *p* < 0.05 from one-way ANOVA with Tukey’s post hoc test, VIP ≥ 1.5, and |*p*(Corr)| > 0.5 from OPLS-DA. RSD > 20% in QC samples were excluded and data were normalized. Other data were analyzed using GraphPad Prism 10.1.2 from GraphPad Software Inc. (Santiago, CA, USA). Results are expressed as mean ± standard deviation (SD). One-way analysis of variance (ANOVA) followed by Tukey’s method comparison test was used to determine significant differences among groups. A *p*-value < 0.05 was considered statistically significant. We thank the reviewer for this helpful comment. We agree that the criteria used to identify differential metabolites should be clearly described.

## 3. Results

### 3.1. Protective Effects of Berberine Against Cadmium-Induced Renal Injury in Mice

**Figure 1:**
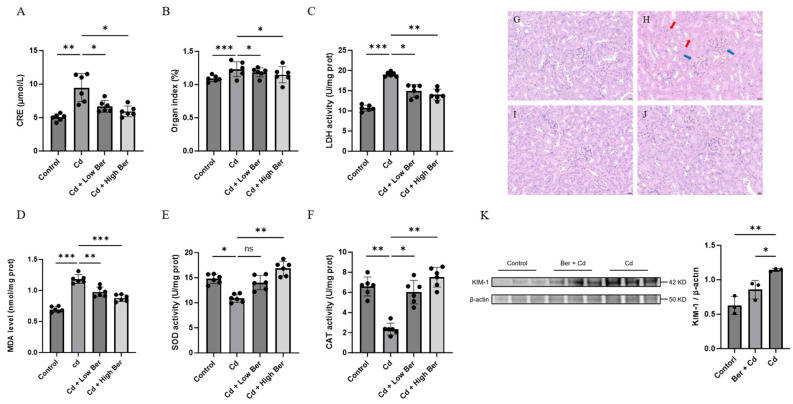
Berberine alleviates cadmium-induced renal injury in mice. (**A**) The effects of Ber supplementation on serum CREA levels of Cd treated mice; (**B**) the effects of Ber supplementation on organ index of kidney of Cd-treated mice; (**C**–**F**) the effects of Ber supplementation on LDH activities, MDA levels, SOD and CAT activities in kidney tissue of Cd treated mice; (**G**–**J**) the effects of Ber supplementation on H- and E-stained kidney tissue in control, Cd-treated, low-dose combination (Cd + low Ber) and high-dose combination (Cd + high Ber) groups of mice. Red arrow, hypercellularity of glomerular cells. Blue arrow, interstitial congestion and edema. (**K**) The effects of Ber supplementation on KIM-1 expression in kidney tissue of Cd treated mice; values normalized to control group (mean ± SD, n = 6), * *p* < 0.05, ** *p* < 0.01, *** *p* < 0.001, ns means not significant, versus the Cd-treated model group. Original western blot images can be found at Supplementary Figure S4.

Ber supplementation decreased organ index compared with the Cd group, indicating reduced kidney edema. Biochemical analysis revealed that Ber significantly lowered CREA levels in Cd-exposed mice. In addition, lactate dehydrogenase (LDH) activity was significantly increased after Cd exposure, whereas Ber treatment markedly restored it to normal levels. Histopathological examination of kidney sections demonstrated that Cd caused renal interstitial congestion, edema, inflammatory infiltration, and marked cellular proliferation within the glomerulus. Analysis of renal tubular injury was performed using the scoring table, and the scoring criteria and result are provided in Supplementary Tables S1 and S2. The results revealed that renal tubules in control group exhibited normal morphology with a score of 0, while high-dose combination showed mild injury with a score of 1.5. Low-dose combination demonstrated moderate injury with a score of 3, and Cd-treated group exhibited the most severe pathological changes with a score of 5, as shown in Figure 1. Overall, a progressive trend in injury severity (G < J < I < H) was observed, indicating an increasing degree of renal tubular injury under the respective experimental conditions. Ber treatment substantially alleviated these alterations in a dose-dependent manner. Western blot analysis further supported the histopathological findings for kidney injury molecule-1 (KIM-1). Cd exposure significance elevated KIM-1 protein expression in mice kidney tissue compared with the control group, reflecting aggravated tubular injury under Cd-induced nephrotoxicity. Ber intervention effectively reversed this increase; Ber treatment reduced KIM-1 protein levels relative to the Cd group, suggesting Ber partial restoration of renal injury. Furthermore, assessment of oxidative stress markers showed that Cd exposure significantly increased MDA levels and decreased SOD and CAT activities compared with controls; these changes were reversed by Ber supplementation. These results indicate that Ber attenuates Cd-induced renal injury and oxidative stress in mice. Moreover, the berberine-only group exhibited no significant changes regarding renal function, histopathological examinations and organ index relative to the normal controls, as shown in Supplementary Materials Figures S1–S3.

### 3.2. Berberine Modulates the Metabolomic Change in Cd-Treated Mice Kidneys

**Figure 2:**
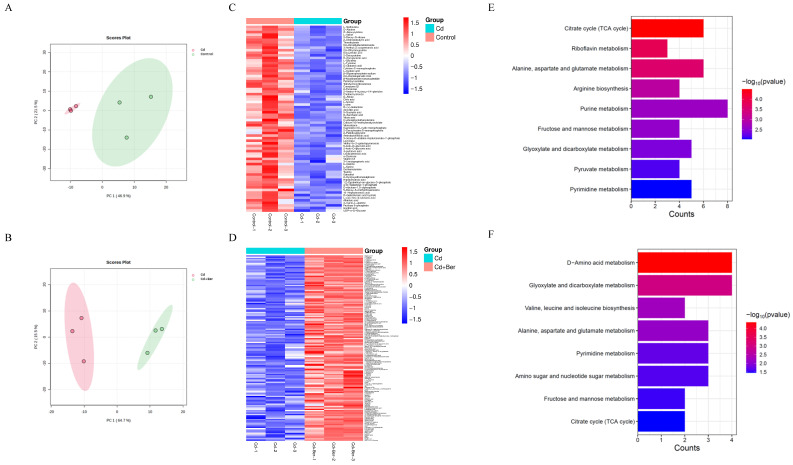
Metabolomics in the kidney tissue of mice. (**A**,**B**) Principal component analysis (PCA) score plot of Cd-treated group versus control group, Cd-treated group versus Cd + Ber group; (**C**,**D**) heatmap of differentially expressed metabolites of Cd-treated group versus control group, Cd-treated group versus Cd + Ber group; (**E**,**F**) KEGG pathway enrichment analysis of differentially expressed metabolites in Cd-treated group versus control group, Cd-treated group versus Cd + Ber group.

Principal component analysis (PCA) was performed to assess the overall metabolic profiles of kidney tissues. Clear separation was observed between the Cd group and the control group, as well as between the Cd group and the combination treatment group, with tightly clustered samples within each group, indicating distinct metabolic shifts between treatments, good experimental reproducibility, and minimal batch effects. As shown in Figure 2A,B, in Figure 2A, PC1 and PC2 explained 46.9% and 21.5% of the total variance, respectively (cumulative 68.4%), while in Figure 2B, PC1 and PC2 explained 64.7% and 15.5% of the total variance, respectively (cumulative 80.2%).

As shown in Figure 2C,D, a total of 73 significantly altered metabolites were identified between the Cd and control groups, whereas 144 metabolites showed significant changes between the Cd and combination treatment groups. Pathway enrichment analysis revealed that the altered metabolites between the Cd and control groups were primarily involved in D-amino acid metabolism, glyoxylate and dicarboxylate metabolism, and valine, leucine, and isoleucine biosynthesis, as shown in Figure 2E. In contrast, the pathways significantly affected between the Cd and combination treatment groups included the TCA cycle, riboflavin metabolism, alanine, aspartate, and glutamate metabolism, as shown in Figure 2F. Moreover, as shown in Supplementary Figure S5, PCA revealed that the berberine-only group largely overlapped with the normal control group, with no significant separation between them.

### 3.3. Berberine Reverses Aberrant Levels of Metabolites

**Figure 3:**
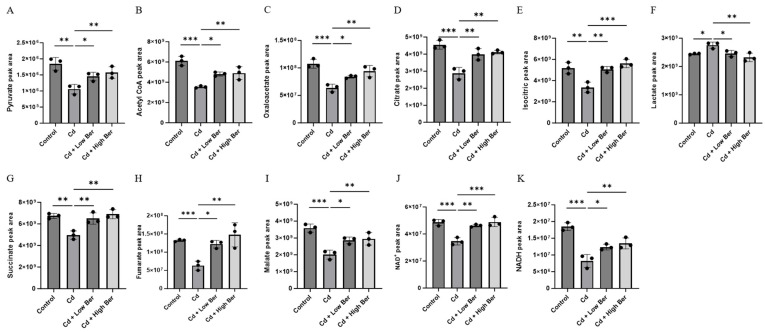
Related metabolites in the kidney tissue of mice. (**A**–**K**) The effects of Ber supplementation on pyruvate, acetyl-CoA, oxaloacetate, citrate, isocitric, lactate, fumarate, succinate, malate, NAD^+^, and NADH levels in kidney tissue of Cd-treated mice; values normalized to control group (mean ± SD, n = 3), * *p* < 0.05, ** *p* < 0.01, *** *p* < 0.001, versus the Cd-treated model group.

To further investigate Ber’s effect on Cd-induced metabolic dysregulation, the levels of key metabolites in the TCA cycle, including pyruvate, acetyl-CoA, oxaloacetate, citrate, isocitrate, fumarate, succinate, malate, as well as the redox cofactors NAD+ and NADH, were quantitatively analyzed. As shown in Figure 3, Cd exposure significantly reduced the levels of all measured metabolites, indicating an overall inhibition of TCA cycle activity and disruption of cellular redox balance.

Specifically, pyruvate, the end product of glycolysis, was significantly decreased in the Cd group, suggesting its accelerated conversion to lactate or diversion into abnormal metabolic pathways. Consistently, lactate levels were markedly elevated in the Cd group, confirming the shift towards anaerobic glycolysis, whereas Ber treatment significantly reduced lactate accumulation in a dose-dependent manner. In the combination treatment groups, pyruvate levels were substantially reversed, indicating that Ber may prevent its excessive depletion.

The Cd group also significantly reduced downstream metabolites acetyl-CoA and oxaloacetate, reflecting potential mitochondrial oxidative dysfunction. Ber treatment significantly increased their levels, with the high-dose combination group showing significant recovery, suggesting improvement in the initial steps of the mitochondrial TCA cycle.

Early TCA intermediates citrate and isocitrate were significantly downregulated after Cd exposure, indicating inhibition of the early steps of the cycle. Ber supplementation normalized metabolite levels close to control, demonstrating its robust metabolic regulatory activity. Furthermore, late-stage TCA intermediates, including fumarate, succinate, and malate, were significantly decreased in the Cd group, suggesting a blockage of the entire cycle and potential impairment of mitochondrial energy supply. Ber treatment induced a dose-dependent elevation in metabolite levels, with high-dose administration driving select metabolites toward physiological control ranges.

In addition, Cd exposure markedly decreased the levels of NAD^+^ and NADH, indicating impaired electron transfer capacity and disturbed mitochondrial redox homeostasis. Ber supplementation significantly restored both NAD^+^ and NADH levels, particularly in the high-dose group, suggesting enhanced mitochondrial function and recovery of cellular energy metabolism. Lactate levels were also measured and showed a significant increase after Cd exposure, which was effectively reversed by Ber treatment.

### 3.4. Berberine Reverses Related Gene Expression

**Figure 4:**
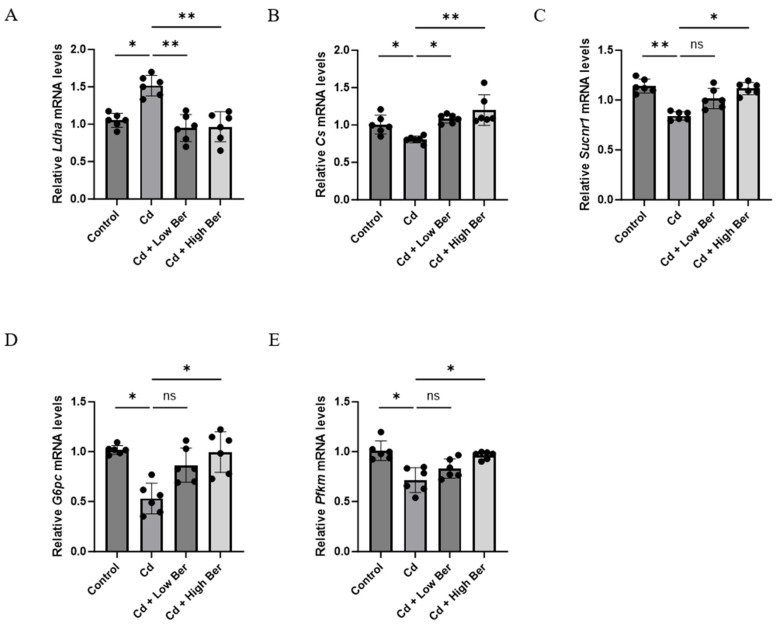
Expression of related mRNA in the kidney tissue of mice. (**A**–**E**) The effects of Ber supplementation on relative mRNA expression levels of *Ldha*, *Cs*, *Sucnr1*, *G6pc* and *Pfkm* in kidney tissue of Cd-treated mice; values normalized to the control group (mean ± SD, n = 6), * *p* < 0.05, ** *p* < 0.01, ns means not significant, versus the Cd-treated model group.

To evaluate the regulatory effects of Ber on Cd-induced metabolic dysregulation, the mRNA expression of key genes involved in renal glucose metabolism was quantified by qPCR. As shown in Figure 4, five genes were selected to represent major metabolic pathways as follows: *Pfkm* and *Ldha* for glycolysis, *Cs* for the TCA cycle, *G6pc* for gluconeogenesis, and *Sucnr1* for metabolite-receptor signaling, thereby providing a transcriptional assessment of metabolic reprogramming in kidney tissue.

Cd exposure significantly upregulated *Ldha* expression relative to the control group, indicating enhanced conversion of pyruvate to lactate and a metabolic shift toward glycolytic dependence. Ber intervention effectively reversed this alteration, as both low- and high-dose treatments significantly reduced *Ldha* mRNA levels. In contrast, Cd markedly suppressed the expression of genes associated with mitochondrial energy metabolism and glucose regulation. Citrate synthase (*Cs*), a rate-limiting enzyme catalyzing the first step of the TCA cycle, was significantly downregulated following Cd exposure. Ber supplementation dose-dependently restored *Cs* expression, with a significant improvement observed in the low-dose group and a more pronounced recovery in the high-dose group.

In addition, the expression levels of succinate receptor 1 (*Sucnr1*), phosphofructokinase (*Pfkm*), and glucose-6-phosphatase (*G6pc*) were significantly reduced in the Cd group. No significant changes were detected after low-dose Ber treatment, whereas high-dose Ber administration significantly increased the expression of all three genes. These findings demonstrate that Ber attenuates Cd-induced renal metabolic disturbances by suppressing aberrant glycolytic activation while restoring TCA cycle function and glucose metabolic homeostasis in a dose-dependent manner.

### 3.5. Berberine Exhibits Strong and Stable Binding to LDHA

**Figure 5:**
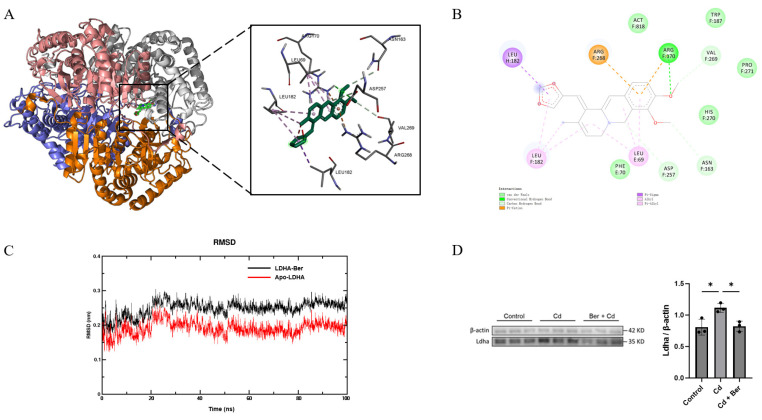
Molecular docking and molecular dynamics simulation of berberine with LDHA and expression of LDHA protein. (**A**) Schematic representation of the binding site of Ber within Ldha; (**B**) two-dimensional interaction map between Ber and LDHA showing hydrogen bonds, hydrophobic contacts, and π interactions; (**C**) root mean square deviation (RMSD) of the Ber–LDHA complex over a 100 ns molecular dynamics simulation, indicating system stability; (**D**) the effects of Ber supplementation on LDHA expression in kidney tissue of Cd-treated mice. * *p* < 0.05, versus the Cd-treated model group. Original western blot images can be found at Supplementary Figure S6.

Molecular docking analysis revealed that Ber binds tightly within the active site of LDHA with a binding energy of −9.515 kcal/mol. The ligand forms conventional hydrogen bonds with ARG170, carbon–hydrogen bonds with ASP257, ASN163, and VAL269, and engages in extensive hydrophobic interactions with nonpolar residues including LEU69, LEU182, PHE70, and TRP187. Additionally, the guanidinium group of ARG268 interacts with aromatic rings via π–cation and π–alkyl interactions, and the C–H edges of the aromatic rings form π–sigma interactions with LEU182, indicating excellent binding affinity, as shown in Figure 5A,B.

To evaluate the effect of berberine binding on LDHA protein dynamics, a 100 ns molecular dynamics (MD) simulation of the LDHA–berberine complex and apo-LDHA was performed under identical conditions. As shown in Figure 5C, apo-LDHA rapidly reached equilibrium and maintained a stable RMSD plateau of approximately 0.20 nm, with only minor fluctuations, indicating that the backbone of unliganded LDHA remained highly stable throughout the simulation. In contrast, the LDHA–berberine complex exhibited a higher RMSD plateau of approximately 0.27 nm after equilibration, representing an approximately 35% increase compared with apo-LDHA. The RMSD increased rapidly during the initial 10–15 ns and stabilized after around 20 ns, fluctuating within a relatively narrow range about 0.05 nm, suggesting that the complex reached equilibrium. The elevated RMSD values of the complex may indicate the occurrence of ligand-induced conformational adjustments or altered structural dynamics upon berberine binding. Importantly, the RMSD trajectory did not show continuous drift and remained within a stable range, indicating that the LDHA–berberine complex maintained overall dynamic stability throughout the 100 ns simulation. Clustering analysis of MD trajectories of the LDHA–berberine complex: we performed additional clustering analysis of the MD trajectory using the GROMOS algorithm with an RMSD cutoff of 0.20 nm. A total of 2667 trajectory frames from the equilibrium stage were analyzed, yielding 10 conformational clusters. The dominant cluster accounted for 64.1% of all analyzed frames, indicating that berberine predominantly maintained a stable binding orientation within the LDHA binding pocket during the simulation. Cluster 2, Cluster 3, and Cluster 4 accounted for 20.4%, 7.8%, and 4.9% of the frames, respectively, whereas the remaining minor clusters collectively represented 2.8% of the total trajectory frames. This distribution indicates that berberine predominantly maintained a stable binding orientation within the LDHA-binding pocket throughout the simulation.

Western blot analysis further supported the transcriptional findings for LDHA. Cd exposure markedly increased LDHA protein expression in mice kidney tissue compared with the control group, indicating enhanced glycolytic activity and accelerated pyruvate-to-lactate conversion under Cd-induced metabolic stress. Ber intervention effectively reversed this alteration, as both low- and high-dose treatment reduced LDHA protein levels relative to the Cd group, suggesting attenuation of abnormal glycolytic activation and partial restoration of metabolic homeostasis.

In conclusion, the low docking energy (−9.515 kcal/mol) and multiple hydrogen bonds, hydrophobic contacts, and π interactions suggest that Ber maintains a stable conformation within the LDHA binding pocket, exhibiting strong binding affinity and dynamic stability.

## 4. Discussion

Cd induces renal toxicity primarily through oxidative stress. Cd accumulates in proximal tubule cells, impairs the electron transport chain, and increases ROS [22,23]. This triggers lipid peroxidation and depletes antioxidants, leading to cellular injury. Numerous studies have reported that Cd elevates kidney MDA levels while reducing SOD and CAT activity [24,25]. We found that Cd-treated mice exhibited elevated CREA, increased renal MDA level and LDH activity, decreased SOD and CAT activity, and severe histopathological changes, consistent with other studies [26]. Ber exerts protective renal effects mainly via its antioxidant properties. In our study, Ber reduced CREA levels and kidney edema. These results align with a meta-analysis showing that Ber significantly lowers CREA in acute kidney injury (AKI) models [27]. Biochemically, Ber-treated mice displayed lower MDA levels and higher SOD and CAT activity compared to Cd controls, indicating reduced oxidative stress. Similar protective effects have been observed in other toxin-induced models. Mombeini reported that Ber reversed cisplatin-induced MDA elevation and restored SOD, CAT, and glutathione peroxidase (GPx) activities [28]. Overall, Ber markedly attenuates the oxidative component of Cd nephropathy and improves renal function.

Our metabolomic analysis revealed that Cd disrupts glycolysis and the TCA cycle in the kidney. Cd-exposed kidneys showed broadly reduced levels of TCA intermediates (citrate, isocitrate, succinate, fumarate, and malate) and substrates (acetyl-CoA, oxaloacetate, and pyruvate), as well as NAD^+^ and NADH, indicating suppression of mitochondrial oxidative metabolism. These findings are consistent with Ilyas’ research, which reported Cd-induced alterations in amino acid and TCA cycle metabolites linked to oxidative stress [29]. Additionally, Cd reduced renal citrate synthase expression, as confirmed by qPCR, while increasing LDHA expression and activity, reflecting a compensatory shift toward glycolysis. Berberine effectively reversed these metabolic disturbances. High-dose Ber restored TCA intermediates and NAD^+^/NADH levels close to those of controls, suggesting recovery of mitochondrial flux. This aligns with Wei’s research, which demonstrated that restoring citrate synthase activity normalized TCA cycle function and protected against AKI. Concurrently, Ber reduced *Ldha* expression, a change that was associated with a metabolic shift away from excessive lactate production. These effects are consistent with prior reports showing that Ber modulates pathological glycolysis.

Our findings indicate renal TCA cycle reprogramming, with succinate and fumarate accumulation alongside altered CS activity. Sadeesh et al. [30] showed that the kidney normally maintains high CS-specific activity to support its energy demand; CS restoration could reflect a return toward this baseline. Martinez-Reyes and Chandel [31] noted that succinate and fumarate act as signaling metabolites that inhibit α-ketoglutarate-dependent dioxygenases, potentially affecting epigenetics and hypoxia responses. In the kidney, SDH expression is typically robust, so succinate build-up may suggest a bottleneck at this step, which might in turn contribute to pseudohypoxia or epigenetic changes. Given that renal tissue relies heavily on oxidative metabolism and tightly controls gluconeogenesis/pyruvate genes [30,32], the observed TCA block and reduced mitochondrial function likely represent a metabolic shift that compromises energy production and may generate pro-pathogenic signals via metabolite depletion. Therefore, strategies aimed at restoring TCA cycle integrity and normalizing CS activity could help re-establish renal homeostasis, a possibility that warrants further validation. Overall, Ber reprograms renal metabolism by alleviating Cd-induced TCA cycle suppression and glycolytic overactivation. However, a limitation of the present study is that CS expression was evaluated solely at the mRNA level by qPCR, and the lack of corresponding Western blot data prevents confirmation of whether the observed changes in CS activity are accompanied by parallel alterations in CS protein abundance.

Cd exposure also downregulated genes involved in mitochondrial function and gluconeogenesis, including *Cs*, *Pfkm*, *G6pc*, and *Sucnr1*. Ber, particularly at high doses, partially restored their expression. Cd-induced suppression of *Sucnr1* and *G6pc* suggests impaired succinate signaling and glucose handling, both of which were improved by Ber. At the same time, Cd upregulated *Ldha*, which Ber reverses. Western blot analysis confirmed these protein-level changes. Mechanistically, Cd enhanced glycolysis and reduced oxidative metabolism, which Ber counteracts. Molecular docking further indicated strong Ber binding to LDHA, forming multiple interactions in the active site and maintaining a stable conformation during simulation. The observed binding affinity, together with the expression data, raises the possibility that modulation of LDHA activity could limit pyruvate-to-lactate conversion and help redirect metabolites toward the TCA cycle; however, these remain correlative observations derived from in silico and expression analyses. Nonetheless, these findings are primarily correlative and derived from molecular docking and expression data, definitive causal evidence that LDHA inhibition directly mediates berberine’s protective effects in vivo requires further validation using LDHA-specific pharmacological inhibitors or genetic knockdown approaches.

In summary, Cd exposure induces ROS accumulation, TCA cycle collapse, and glycolytic shift, resulting in energy deficiency and renal injury. Berberine mitigates these effects by scavenging ROS, enhancing antioxidant defenses (SOD/CAT), modulation of LDHA-associated glycolytic pathways, and restoring TCA enzyme activity, collectively protecting kidney function. However, we cannot fully rule out direct effects of berberine on renal under normal conditions. Further studies with such a control group are needed to confirm the disease-specific nature of the protective effects observed here.

## 5. Conclusions

In conclusion, berberine effectively mitigated cadmium-induced nephrotoxicity in mice, as evidenced by improved renal function, reduced histopathological damage, lower lipid peroxidation and tissue injury, and recovery of endogenous antioxidant defenses. Mechanistically, cadmium exposure was associated with a shift characterized by LDHA upregulation, depletion of pyruvate and tricarboxylic acid cycle intermediates, and disruption of NAD+/NADH homeostasis, whereas berberine reversed these abnormalities at the metabolite, transcriptional, and protein levels. Molecular docking and dynamics analyses further support LDHA as a possible interacting protein that may contribute to the metabolic effects of berberine. Overall, berberine protects against Cd-induced renal injury through coordinated antioxidant and metabolic reprogramming actions, particularly by influence the LDHA-driven glycolytic shift and restoring mitochondrial oxidative metabolism. These findings provide a mechanistic basis for further preclinical evaluation of berberine as a potential therapeutic agent for cadmium-related kidney injury.

## Data Availability

The data that support the findings of this study are available from the corresponding author, Shusheng Tang, upon reasonable request.

## Supplementary Materials

The following supporting information can be downloaded at https://www.mdpi.com/article/10.3390/biom16070951/s1, Figure S1: Renal injury score result; Figure S2: The effects of Ber supplementation on serum CREA levels; Figure S3: The effects of Ber supplementation on organ index of kidney; Figure S4: Original western blot image of Figure 1K; Figure S5: Principal component analysis (PCA) score plot comparing the control and Ber groups; Figure S6: Original western blot image of Figure 5D; Table S1: Renal injury scoring table; Table S2: Renal injury score result table.
