# Supplementary material for: Berberine Attenuates Cadmium-Induced Nephrotoxicity by Suppressing LDHA-Mediated Glycolytic Reprogramming and Restoring Mitochondrial TCA Cycle Metabolism
Source: Biomolecules. 2026 Jun 26;16(7):951. doi: 10.3390/biom16070951 (PMC13407037; doi:10.3390/biom16070951)
Supplement: Supplementary file 1 [file biomolecules-16-00951-s001.zip › biomolecules-4373778-supplementary.pdf]

Berberine Attenuates Cadmium-Induced Nephrotoxicity by Suppressing LDHA-Mediated Glycolytic Reprogramming and Restoring Mitochondrial TCA Cycle Metabolism

Zikang Zeng ,Weidong Qiao , Yuanyuan Zhang and Shusheng Tang

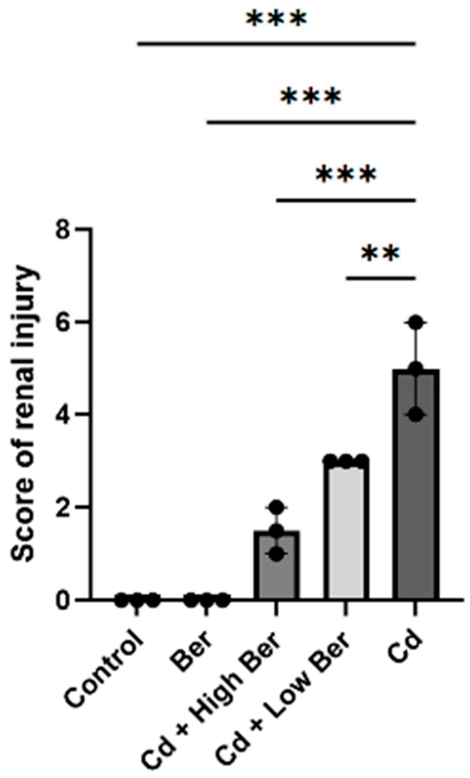

Figure S1. Renal injury score result.

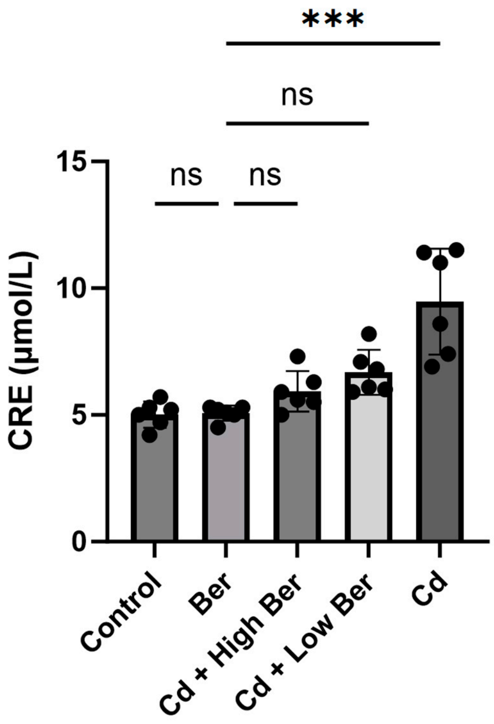

Figure S2. The effects of Ber supplementation on serum CREA levels.

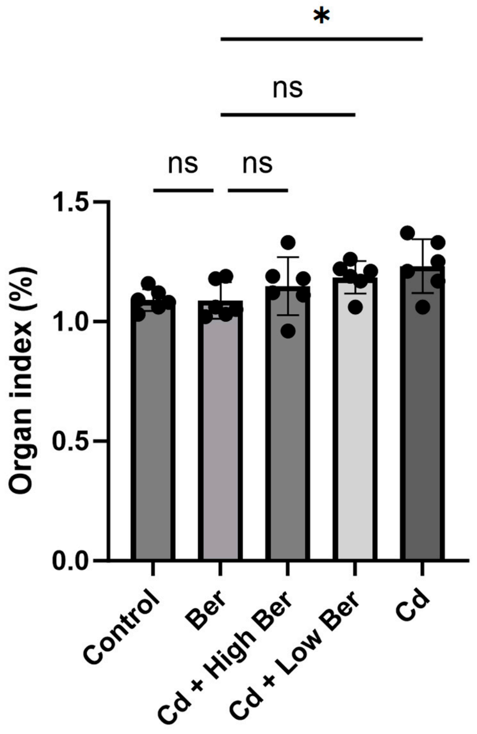

Figure S3. The effects of Ber supplementation on organ index of kidney.

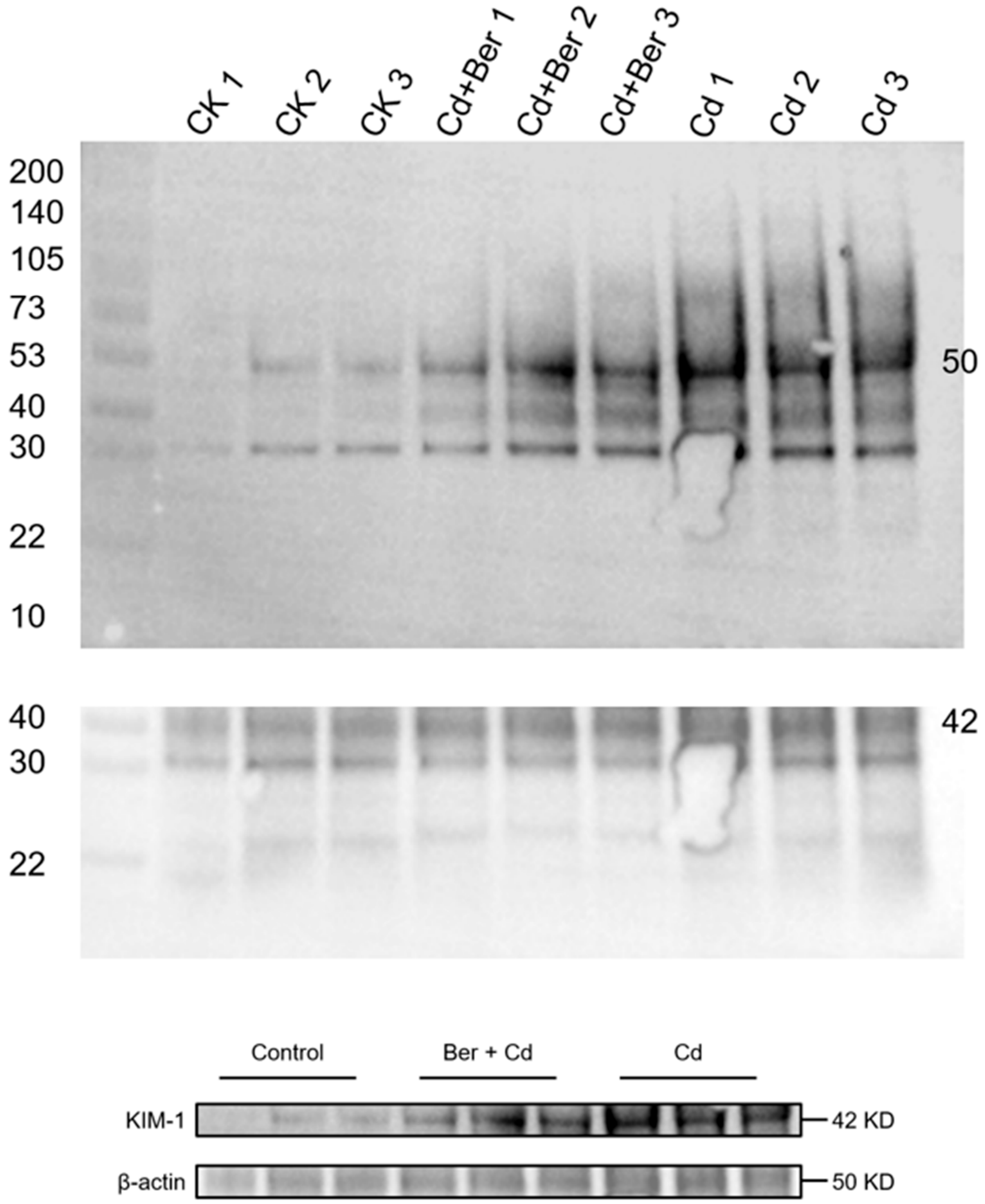

Figure S4. Original western blot image of Figure 1K.

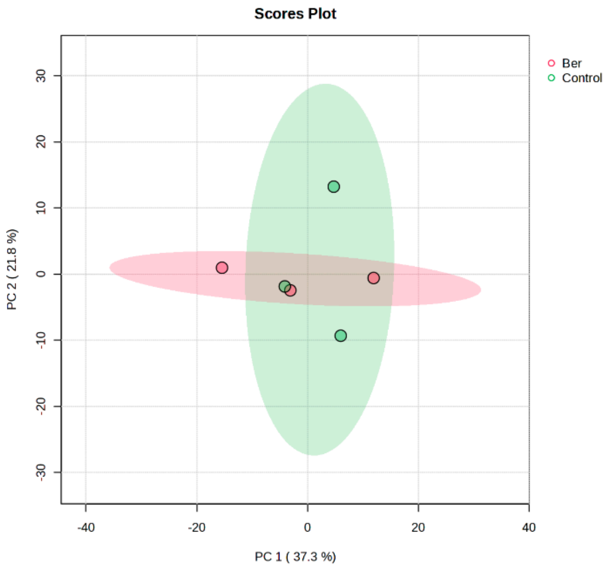

Figure S5. Principal component analysis (PCA) score plot comparing the control and Ber groups.

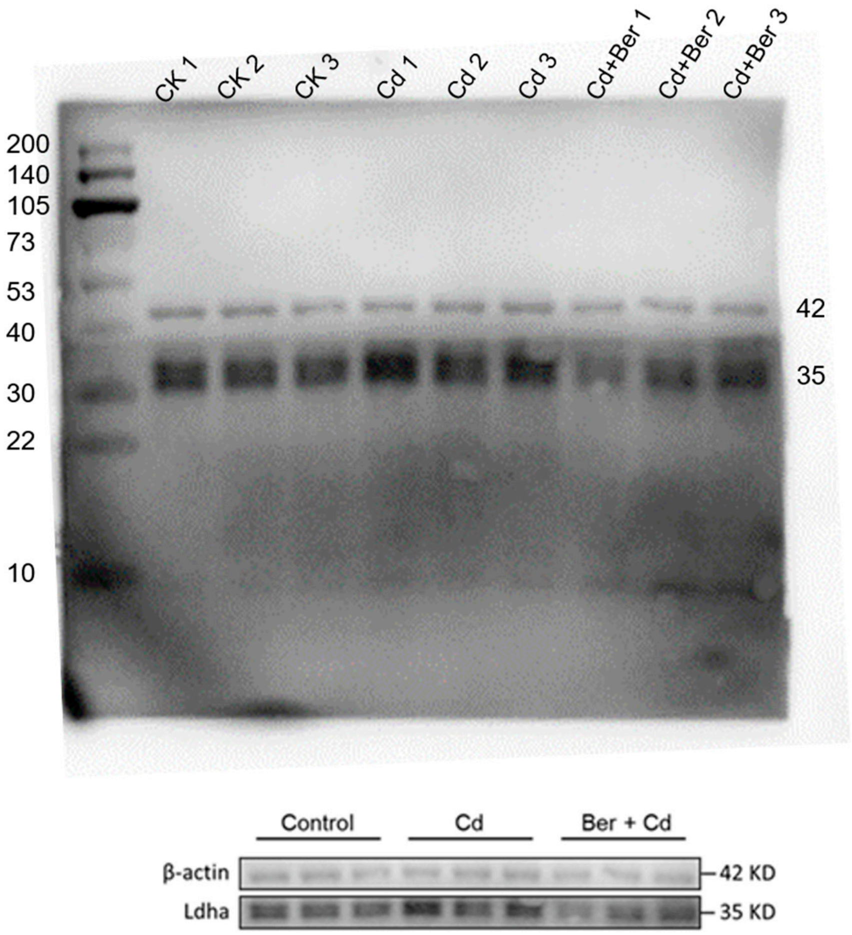

Figure S6. Original western blot image of Figure 5D.

Table S1. Renal injury scoring table.

Category	Description	Score
Degeneration or Necrosis	None	0
Degeneration or Necrosis	Scattered	1
Degeneration or Necrosis	Majority	2
Degeneration or Necrosis	Almost All	3
Degeneration or Necrosis	All	4
Vacuolization	None	0
Vacuolization	Scattered	0.5
Vacuolization	Majority	1
Vacuolization	Almost All	1.5
Vacuolization	All	2
Swelling	None	0
Swelling	Scattered	0.5
Swelling	Majority	1
Swelling	Almost All	1.5
Swelling	All	2

Table S2. Renal injury score result table.

Group	Scores	Mean ± SD
Control	0,0,0	0.00 ± 0.00
Ber	0,0,0	0.00 ± 0.00
Cd + High Ber	2,1.5,1	1.50 ± 0.50
Cd + Low Ber	3,3,3	3.00 ± 0.00
Cd	5,4,6	5.00 ± 1.00
